# Case Report: Renal hemangiosarcoma in a free-ranging red fox (*Vulpes*vulpes)

**DOI:** 10.3389/fvets.2026.1766425

**Published:** 2026-02-27

**Authors:** Adriano Minichino, Giuseppina Mennonna, Maria Dimatteo, Barbara degli Uberti, Marianna D'Amore, Francesca Santomartino, Evaristo Di Napoli, Orlando Paciello, Luigi Maria De Luca Bossa, Ludovico Dipineto, Luca Borrelli, Guido Rosato

**Affiliations:** 1 Wildlife Rescue and Rehabilitation Center (CRAS), Napoli, Italy; 2 Istituto Zooprofilattico Sperimentale del Mezzogiorno, Portici, Italy; 3 Department of Veterinary Medicine and Animal Production, University of Naples Federico II, Naples, Italy; 4 Centro Regionale per l'Igiene Urbana Veterinaria (CRIUV), Naples, Italy

**Keywords:** hemangiosarcoma, red fox, renal tumor, wildlife pathology, wildlife surveillance

## Abstract

This report describes the first documented case of primary renal hemangiosarcoma in a male red fox (*Vulpes vulpes* Linnaeus, 1758) admitted to the Wildlife Rescue Center “Federico II”–Veterinary Hospital of the Local Health Authority (ASL) Napoli 1 Centro in Naples, Italy. The fox, originating from a sparsely urbanized area, presented with progressive debilitation. Diagnostic imaging (ultrasound, radiography, CT) identified a mass in the right kidney. Citology suggested a malignant neoplasm, and histopathology confirmed hemangiosarcoma with multiple metastases to abdominal organs and to the heart. Necropsy revealed splenomegaly and a bladder lesion, while virological and bacteriological investigations excluded infectious diseases. The internal abdominal hemorrhages as consequence of tumor rupture were considered the cause of hypovolemic shock. This case underscores the value of advanced diagnostic techniques and systematic post-mortem investigations in wildlife medicine, contributing to the limited knowledge of neoplastic diseases in free-ranging foxes and highlighting the importance of wildlife health surveillance for ecological insight.

## 1 Introduction

Hemangiosarcoma (HSA) is a malignant tumor originating from vascular endothelial cells, characterized by rapid growth, aggressive biological behavior, and a high metastatic potential (1). This neoplasm represents approximately 2% of all canine tumors and it is overrepresented in specific breeds such as German Shepherds, Golden Retrievers, and Labrador Retrievers (1, 2). While most frequently observed in the spleen, right atrium of the heart, liver, and skin, HSA can also develop in less common sites, including the kidneys, retroperitoneum, central nervous system, and urinary tract (3, 4). In dogs, the clinical presentation is often no specific, with signs such as lethargy, anorexia, and acute collapse depending on the tumor location and stage (5).

Despite its frequent occurrence in domestic animals, cases involving wildlife are rarely documented, highlighting a critical gap in understanding this neoplasm in free-ranging species (6, 7). Advanced imaging modalities such as ultrasonography, computed tomography (CT), and magnetic resonance imaging (MRI) are essential for diagnosing and staging HSA in animals. These techniques allow clinicians to evaluate tumor size, vascular involvement, and potential metastases (6, 8). However, imaging findings alone are often insufficient to differentiate malignant HSA from benign masses such as hemangiomas or hematomas (3, 5). Definitive diagnosis relies on histopathological examination, supported by immunohistochemical markers such as Vimentine and specifically vascular markers such as factor VIII, CD31 (PECAM), and CD34 (4, 9–11). The biological behavior and prognosis of HSA vary significantly based on the tumor's primary location. For instance, heart and splenic HSA is associated with a high metastatic rate (16%−89%) and a median survival time (MST) of less than 3 months following surgical intervention (3, 5). Conversely, renal and retroperitoneal HSA exhibit lower metastatic rates and comparatively better survival times when managed surgically, with median survival exceeding 200 days in some cases (3, 6). Tumor size, invasion into adjacent tissues, and the presence of metastases significantly influence prognosis (2, 9). This variability underscores the importance of detailed diagnostic and staging protocols to guide clinical decision-making. In wildlife, HSA is not reported in red fox, and the available literature on neoplasms is limited to isolated case studies in this species (12–14). Neoplastic diseases in wild species may serve as valuable indicators of environmental health, reflecting exposure to contaminants, habitat degradation, or other stressors (15). As apex predators or sentinel species, wild carnivores such as the red fox (*Vulpes vulpes*) are particularly important for monitoring ecosystem dynamics and health (7, 12–15). This report documents the first known case of primary renal hemangiosarcoma in a red fox, combining advanced diagnostic imaging, cytological evaluation, and histopathological analysis. The fox originated from a sparsely urbanized area in southern Italy and presented with clinical signs of renal impairment. This case not only broadens the understanding of hemangiosarcoma in wild carnivores but also emphasizes the critical role of wildlife health surveillance in assessing ecological stability and addressing conservation challenges.

## 2 Case description

An adult male red fox (*Vulpes vulpes*, Linnaeus, 1758), was admitted on October 28, 2024, to the Wildlife Rescue Center (CRAS) “Federico II” in Naples, at the Veterinary Hospital of the Local Health Authority (ASL) Napoli 1 Centro, after being found in a sparsely urbanized area. The fox showed depression and posterior paraparesis. Clinical examination revealed normal temperature and pulse, pink mucous membranes, and mild dehydration. There was a mild nociceptive deficit in the hind limbs and pain upon abdominal palpation, suggesting possible intra-abdominal pathology. The fox was sedated with an intramuscular combination of medetomidine (30 μg/kg) and ketamine (5 mg/kg), followed by the placement of a 22-gauge venous catheter and administration of isotonic fluids (Ringer's lactate at 50 ml/kg). Following clinical deterioration, the animal died during hospitalization and was subsequently submitted for post-mortem examination.

### 2.1 Radiography

At the Diagnostic Imaging Department of the Veterinary Hospital of the Local Health Authority (ASL) Napoli 1 Centro, the patient underwent an X-ray examination of the thoracolumbar spine in latero-lateral (LL) projection, in right recumbency, and in ventrodorsal (VD) projection. The X-ray study did not reveal any lesions, except for a slight reduction in the T13–L1 intervertebral space. For this reason, the patient subsequently underwent a CT scan of the spine.

### 2.2 Computed tomography

**Figure 1:**
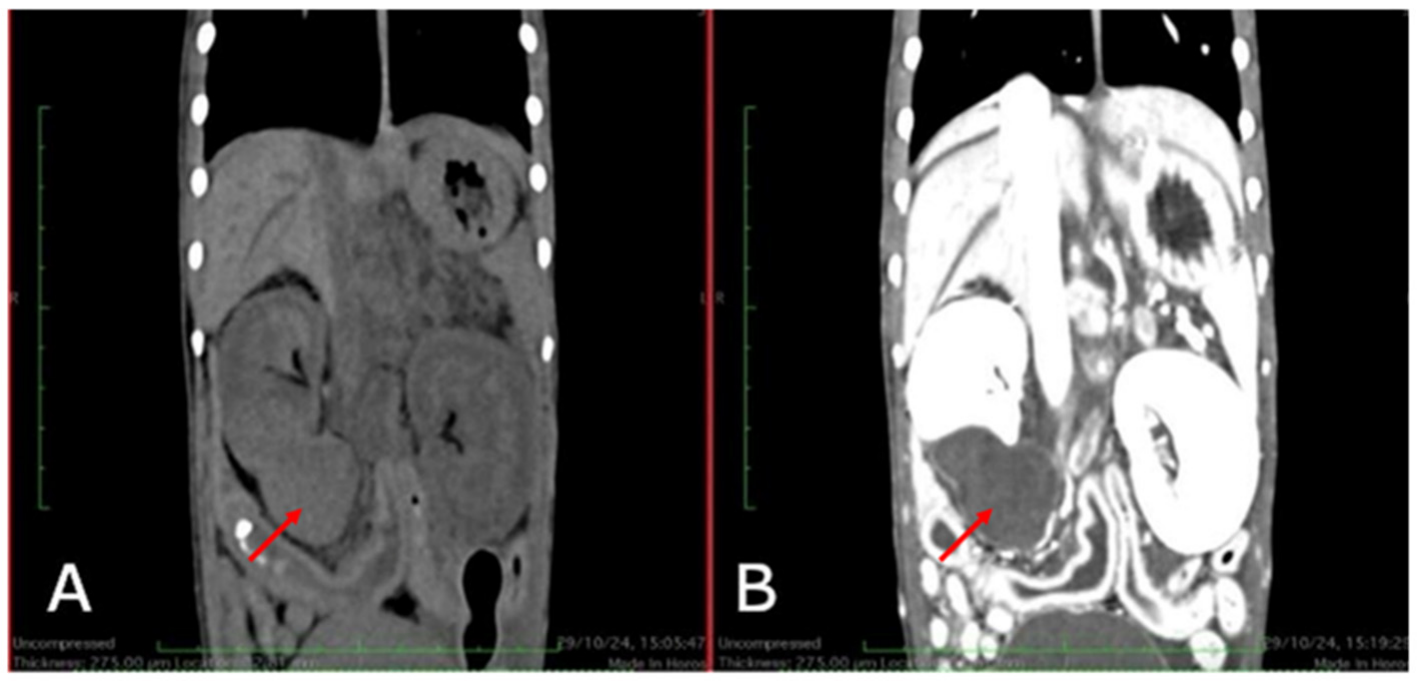
Coronal CT image: **(A)** Pre-contrast; **(B)** Post-contrast nephrographic phase. In the pre-contrast image, a heterogeneous appearance of the right kidney is observed due to an area of hypoattenuation (red arrow). Following contrast medium administration, heterogeneous enhancement is evident, primarily exhibiting a ring pattern (red arrow).

The CT scan was performed using an 80-slice multidetector scanner (Aquilion Prime, Canon Medical Systems Corporation, Tochigi, Japan) with helical scanning and thin-slice reconstructions (0.5 mm) using soft tissue and bone filters. The Field of View (FOV) was adjusted according to the region of interest, and the scan was conducted with parameters of 120 kV and 140 mAs, with a rotation time of 0.5 s. The subject was positioned in a dorsal recumbency as the region of interest was the spine. Scans were acquired before and after the intravenous administration of a contrast agent, Iopamidol (Iopamiro^®^ 370 mg/ml injectable solution, BRACCO IMAGING, Milan, Italy), at a dose of 2 ml/kg over 1 min. Post-contrast scans were obtained in both arterial and venous phases. In the pre- contrast scans, a large expansive lesion was evident in the caudal pole of the right kidney, showing loss of normal architecture and a heterogeneous appearance with slightly hypoattenuating areas (Figure 1). Following contrast administration, an enhancement was predominantly ring-shaped.

### 2.3 Ultrasonography

**Figure 2:**
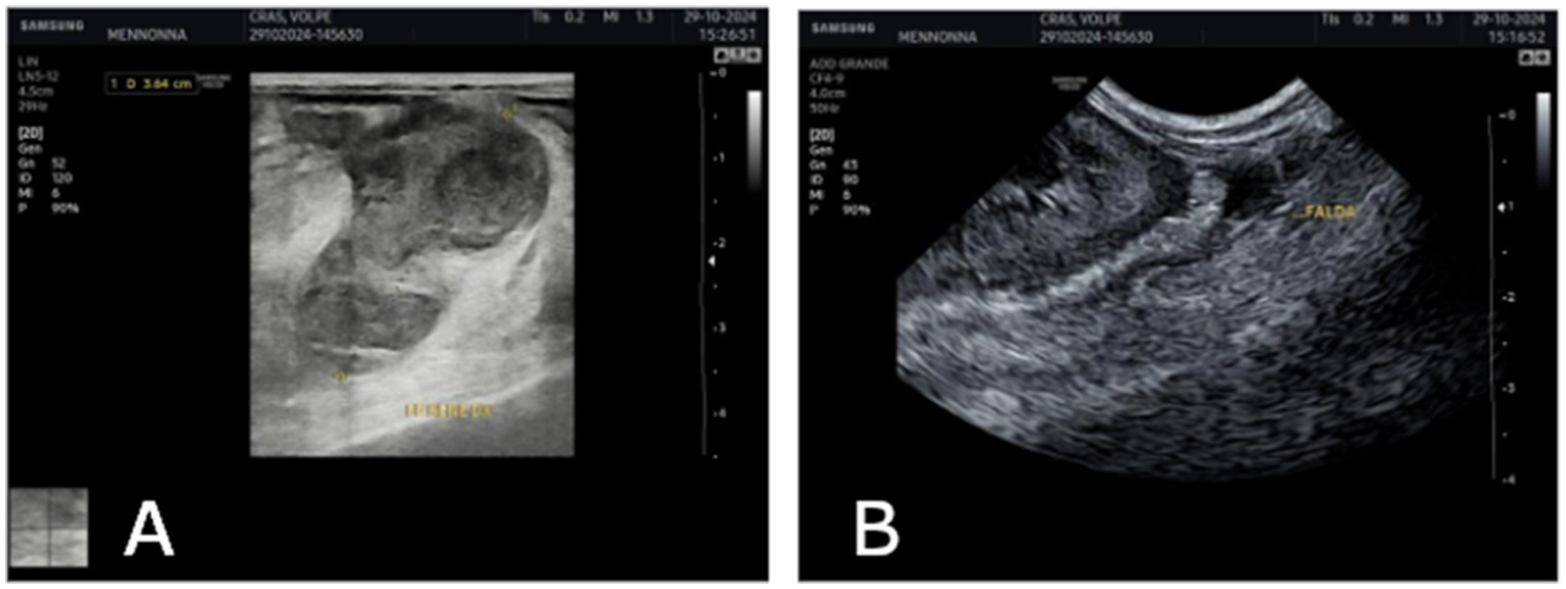
The ultrasound image **(A)** caudal pole of the right kidney shows a complete alteration of the normal echotexture due to the presence of a large expansive mass with heteroechogenic, characterized by both hyperechoic and ipoechoic areas. **(B)** peritoneal effusion.

An abdominal ultrasound was subsequently performed using a Samsung HS30 ultrasound machine (Samsung Healthcare, Seoul, Republic of Korea) with a microconvex probe operating at a multifrequency range of 4.0–9.0 MHz. The fox was positioned in dorsal recumbency, and the fur on the abdomen was removed. The examination was conducted in real-time B-mode. The kidney was scanned in longitudinal-dorsal and transverse planes. Ultrasound findings revealed that the expansive lesion in the caudal pole of the right kidney exhibited complex echogenicity, with hypo-/anechoic areas as well as echogenic/isoechoic areas compared to the renal parenchyma. A small amount of anechoic retroperitoneal effusion was also observed (Figure 2).

### 2.4 Citology

The citological examination obtained through ultrasound-guided 22-gauge needle aspiration of the mass showed moderate cellularity, with a good staining pattern and conservation of citological details, although interpretation is partially limited by marked blood contamination. It consists mainly of a population of mesenchymal-appearing cells arranged singly or in small, loosely cohesive aggregates. These cells are fusiform or oval in shape, approximately 1.5–2 times the diameter of an erythrocyte, with scant, weakly basophilic cytoplasm and indistinct cell margins. The nuclei are oval, variable in size, sometimes eccentric, with finely dispersed chromatin and occasionally prominent nucleoli. Marked anisocytosis and anisokaryosis are present, with moderate to marked nuclear pleomorphism, and mitotic figures, including atypical forms, are observed. The background contains abundant blood, numerous well-segmented neutrophils, rare activated foamy macrophages, even rarer lymphocytes, and small amounts of amorphous necrotic debris (Supplementary figures). Overall, the findings are consistent with malignant mesenchymal proliferation, most compatible with a soft tissue sarcoma.

### 2.5 Necropsy

The cadaver was sent to the Istituto Zooprofilattico del Mezzogiorno (IZSM) for necropsy and histological analysis. Necropsy revealed normotrophic muscle mass, although the animal appeared lean.

**Figure 3:**
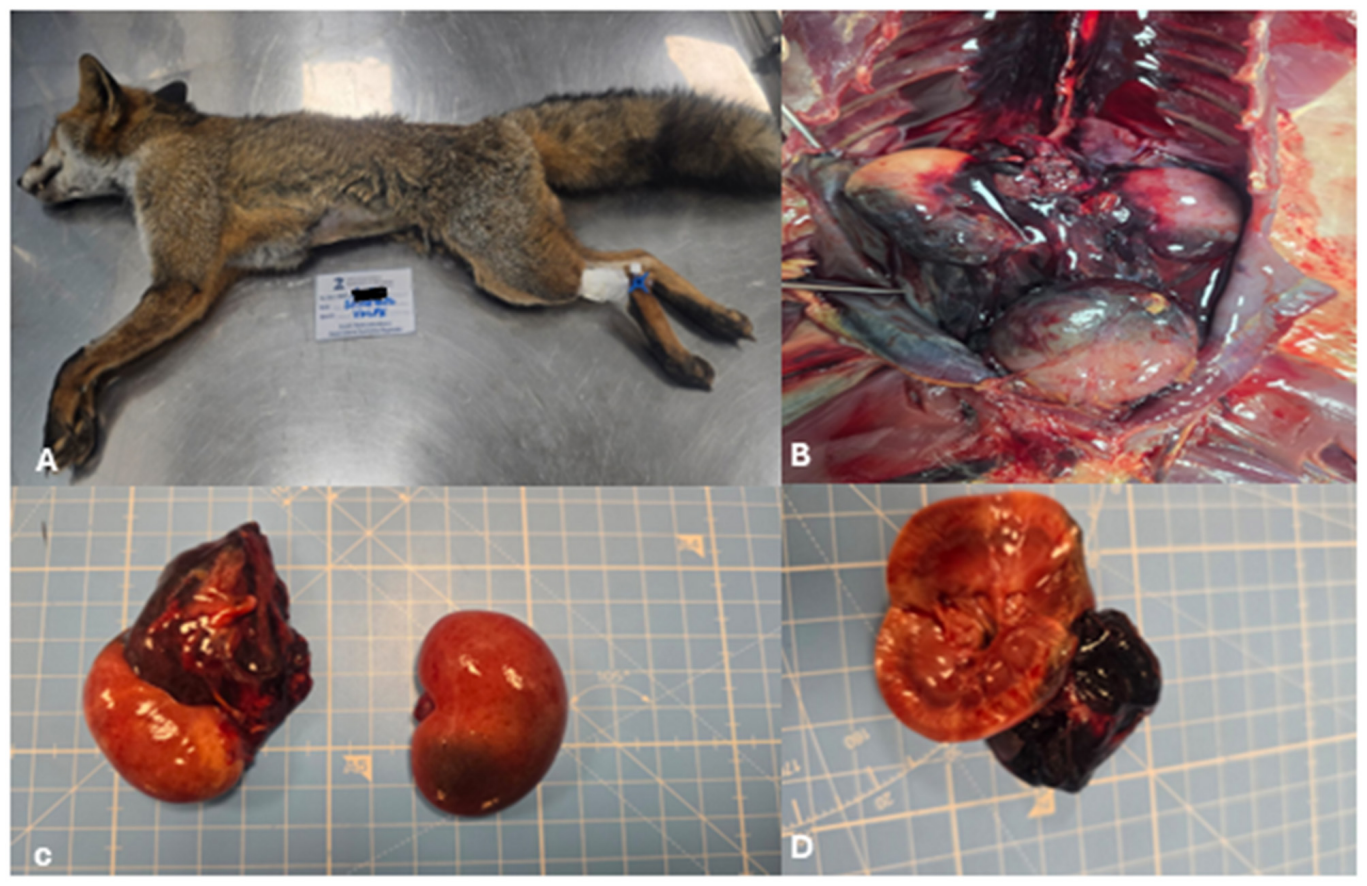
Macroscopic findings observed during the necropsy of a red fox (*Vulpes vulpes*). **(A)** External view of the cadaver before opening the body cavities, showing an adult male in lean condition with well-developed muscle masses. **(B)** Kidneys in situ within the abdominal cavity: an irregular, reddish-brown extracapsular mass is visible at the cranial pole of the right kidney. **(C)** Gross appearance of the isolated right kidney, showing a protruding lesion with poorly defined, infiltrative margins. **(D)** Sectioned right kidney: the mass appears hemorrhagic with infiltrative borders, disrupting the corticomedullary architecture and suggesting a neoplastic process.

Subcutaneous structures were examined, revealing a poorly developed adipose panniculus with no evidence of hematomas or ecchymoses. In the abdominal cavity, the necropsy noted an abundant hemorrhagic effusion with hemoabdomen, while the intracavitary organs were in their physiological positions. The liver appeared normal in shape and size, with a mottled parenchyma characterized by pronounced perilobular patterns and alternating light and dark areas. Splenomegaly was observed, with the spleen showing a uniform brown coloration and retained pulp on the cut surface. At the lateral edge of the cranial pole of the right kidney, the necropsy identified an extracapsular, irregularly shaped, reddish-brown mass. This lesion protruded and had infiltrative, poorly defined margins and was blood-filled, suggesting a neoplastic process (Figure 3). The contralateral left kidney retained its shape and volume, presenting a smooth surface and reddish-brown coloration, with evident corticomedullary differentiation at the opening. The bladder was distended, extending cranially in the abdomen, and a focal, irregular reddish-brown mass was identified at the bladder neck. This mass displayed poorly defined margins and extensive areas of necrosis and hemorrhage. The bladder wall was thickened, and the urine appeared clear upon sectioning. In the thoracic cavity, the heart was normal in shape and size, with preserved chamber ratios. Coagulated blood was present in the atrioventricular chambers, and no pericardial effusion was observed. A focal, dark reddish-purple lesion, a few millimeters in diameter, was present on the right atrium epicardium. The lungs exhibited severe congestion and atelectasis, with a smooth external surface of reddish-purple coloration. Hemorrhages were noted in both caudal lobes, while the trachea was patent and free of luminal contents. The gross examination of the other internal organs revealed no other lesions.

### 2.6 Histology

**Figure 4:**
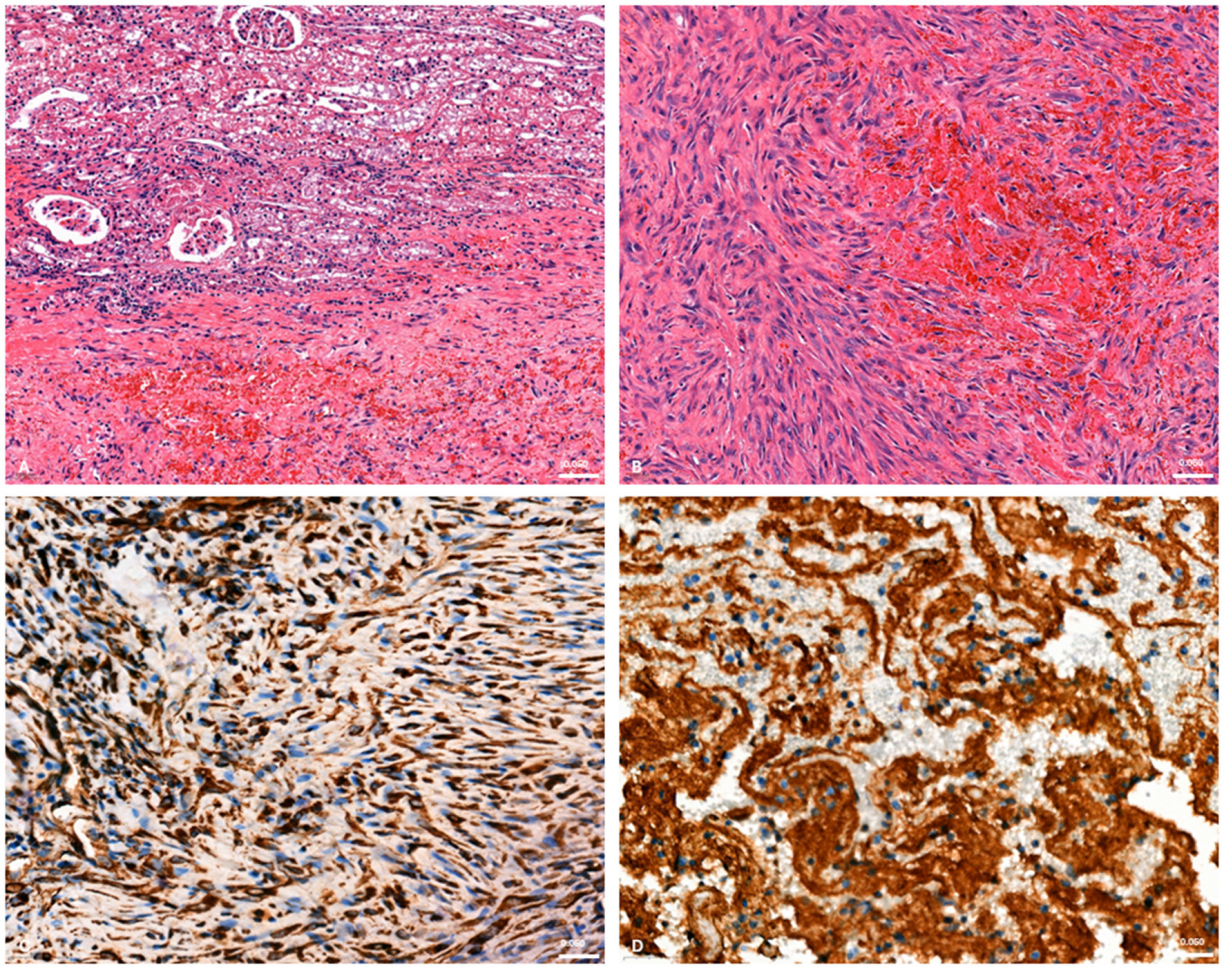
Histological section of right kidney with mesenchymal neoplastic cell proliferation miming vascular channels **(A)** and extracapsular neoplastic invasion of the hemorrhagic peritoneum **(B)** with spindle cells organized in whorls and streams. (Hematoxylin and eosin, 20×); Immunohistochemistry in kidney section showing intracytoplasmic immunopositivity to Vim **(C)** and vWf **(D)** antibodies in neoplastic cells (40×).

Both kidneys, bladder, heart, liver, lung, spleen, small intestine and its lymph node and brain were collected and fixed in 10% neutral buffered formalin for histological examination. Tissue samples were trimmed and embedded in paraffin. Microsections 3–4 μm thick were routinely stained with haematoxylin and eosin with a standard protocol (Leica Autostainer XL, Heidelberger, Germany). For immunohistochemistry (IHC), additional sections of the renal tumor samples (3–4 μm thick) were processed with the Bond Polymer Refine Detection Kit (Leica Biosystem, Nussloch, Germany). The primary antibodies were Vimentine (Vim) and Factor VIII-related antigen (vWf) ready to use. The slides were automatically stained by means of Leica Bond III immunostainer (Melbourne, Australia) and were, then, digitalized with Pannoramic 250 Flash III scanner (Budapest, Hungary) to take photographs. Histological examination of the urinary tract revealed an unencapsulated, poorly circumscribed and infiltrating neoplasm that extended from renal hilum to the cortex. Neoplastic proliferation partially involved the submucosa and muscularis, with extension into the adventitia and adjacent peritoneal serosa of urinary bladder neck and ureters. Neoplastic cells were spindle-shaped forming irregular vascular channels filled with blood and streams and tightly packed whorls surrounding and entrapping glomeruli and tubules. They have indistinct cell borders, a scant amount of cytoplasm and nucleus with vesiculate clumped chromatin and prominent multiple nucleoli. The tumor cells exhibited marked anisocytosis and anisokaryosis. Mitotic count is variably high with 10 mitoses in 2.37 mm^2^ (16) (Figures 4a, b). The left kidney showed no neoplastic lesions. Metastatic lesions were also identified infiltrating the peritoneum and adjacent organs such as spleen, intestinal wall and its related lymph node demonstrating similar histological characteristics consistent with metastatic hemangiosarcoma. Micrometastasis was observed in the right atrium of the heart where neoplastic cells induced hemorrhages in the epicardium. Immunohistochemical analysis was performed on the right kidney section showing a strong diffuse cytoplasmic immunoreactivity of the most neoplastic cells for Vimentine (Figure 4c) and a strong diffuse cytoplasmic immunoreactivity for factor VIII (Figure 4d), normally expressed in the endothelial cells. In brain, lung and liver no neoplastic lesions were observed.

### 2.7 Infectious and parasitic disease investigations

Virological examinations, including Real-Time PCR for suid herpesvirus 1 (SHV-1) and rabies virus (RABV) testing through cell culture isolation and nucleocapsid antigen detection via direct immunofluorescence (OIE Manual for Terrestrial Animals 2011, Chapter 2.1.13), yielded negative results. Bacteriological and parasitological analysis did not detect any pathogen.

## 3 Discussion

This case report describes the first documented occurrence of primary renal hemangiosarcoma in a free-ranging red fox and contributes to the limited body of literature on neoplastic diseases in wild carnivores. In dogs, hemangiosarcoma is well recognized as an aggressive vascular neoplasm with a predilection for the spleen, right atrium, liver and, less frequently, the kidneys. Renal hemangiosarcoma is considered an uncommon localization even in this species, and data on its biological behavior are relatively scarce compared with splenic or cardiac forms. The present case shows that, in the red fox, renal hemangiosarcoma may follow a highly aggressive clinical course, characterized by extensive intra-abdominal dissemination. The clinical presentation was nonspecific, with generic signs that preceded acute decompensation due to hemorrhage or rupture of the mass. Advanced diagnostic imaging techniques played a central role in the characterization of the renal lesion. Ultrasonography revealed a large heterogeneous mass replacing the caudal pole of the right kidney, with mixed echogenicity and associated fluid accumulation. Computed tomography further defined the lesion as a voluminous, heterogeneously hypoattenuating mass with peripheral or ring-like contrast enhancement. On contrast-enhanced CT, the spleen showed a small hypodense area with moderate enhancement, no appreciable abnormalities were detected in the heart, and an enlarged lymph node was observed with regular and homogeneous contrast enhancement. In dogs, such imaging features are often associated with malignant vascular tumors, large hematomas or necrotic neoplasms, and do not by themselves allow hemangiosarcoma to be distinguished from other malignant renal tumors, such as sarcomas, carcinomas or lymphomas. Nonetheless, cross-sectional imaging allowed accurate localization of the lesion, assessment of the degree of renal involvement and identification of abdominal effusion, providing key elements for clinical decision-making and for planning the pathological investigation. Cytological examination of the mass yielded findings consistent with a malignant mesenchymal neoplasm. The predominance of spindle to oval cells with marked anisocytosis and anisokaryosis, prominent nucleoli and occasional mitotic figures on a strongly hemorrhagic background is in line with the cytological appearance of vascular sarcomas in domestic species. However, as frequently reported for hemangiosarcoma, marked hemodilution and lesion friability limited the diagnostic yield, precluding definitive classification. This underscores a well-known limitation of fine-needle aspiration in vascular tumors, in which histopathology and immunohistochemistry remain indispensable for an accurate diagnosis. Histologically, the tumor was a non-encapsulated, infiltrative neoplasm extending from the renal hilus to the cortex, with invasion of the ureteral and bladder wall. The neoplastic cells formed irregular blood-filled vascular channels and compact whorls, and showed marked pleomorphism and a high mitotic index, all features typical of malignant endothelial neoplasms. Immunohistochemical expression of Vimentin and von Willebrand factor (factor VIII– related antigen) in neoplastic cells confirmed their mesenchymal and endothelial nature, supporting the diagnosis of hemangiosarcoma. The presence of similar neoplastic proliferations in the peritoneum, spleen, intestinal wall, associated lymph node and at the level of the right atrial epicardium indicates widespread metastasis, mirroring the metastatic pattern described in canine hemangiosarcoma and highlighting the highly malignant behavior of this tumor in the red fox. The metastatic lesion observed on the epicardium of the right atrium is particularly noteworthy. In dogs, primary cardiac hemangiosarcoma classically arises in the right atrium or auricle and may coexist with, or metastasize to, abdominal organs. In the present case, the small epicardial focus was histologically and immunohistochemically compatible with a metastasis from the primary renal tumor rather than a distinct primary cardiac lesion. This finding illustrates the ability of renal hemangiosarcoma to disseminate to typical target sites of vascular neoplasms and reinforces the need for accurate necropsy with systematic sampling of multiple organs in cases involving wildlife. From an ecological and epidemiological perspective, neoplastic diseases in free-ranging wildlife remain underestimated and underreported, mainly due to limited diagnostic resources, the absence of systematic necropsy programs and the perception that tumors are less relevant than infectious or toxicological hazards. This case helps to partially fill this gap by documenting a malignant renal neoplasm in a synanthropic carnivore and demonstrating that advanced diagnostic approaches commonly used in companion animals can also be successfully applied to wildlife. The involvement of the urinary tract and the extensive peritoneal dissemination further raise questions about the possible role of environmental or toxic cofactors, although no specific etiological agents were identified in this animal, the case underscores the importance of integrating tumor surveillance into broader environmental health assessment systems. This report has some limitations inherent to descriptive studies on single cases. The absence of long-term clinical follow-up, the inability to perform comprehensive toxicological analyses and the lack of comparable cases in the same area limit the possibility of formulating causal hypotheses or drawing conclusions at the population level. Nevertheless, the detailed clinical, imaging and pathological characterization, combined with integration into an established surveillance network, demonstrates the feasibility and added value of intensive diagnostic investigation even in wild animals.

## Data Availability

The original contributions presented in the study are included in the article/Supplementary material, further inquiries can be directed to the corresponding author.
