# Supplementary material for: Case Report: Renal hemangiosarcoma in a free-ranging red fox (Vulpesvulpes)
Source: Front Vet Sci. 2026 Feb 27;13:1766425. doi: 10.3389/fvets.2026.1766425 (PMC12983464; doi:10.3389/fvets.2026.1766425)
Supplement: Supplementary file 1 [file Table_1.docx]

**Supplementary figures**

**
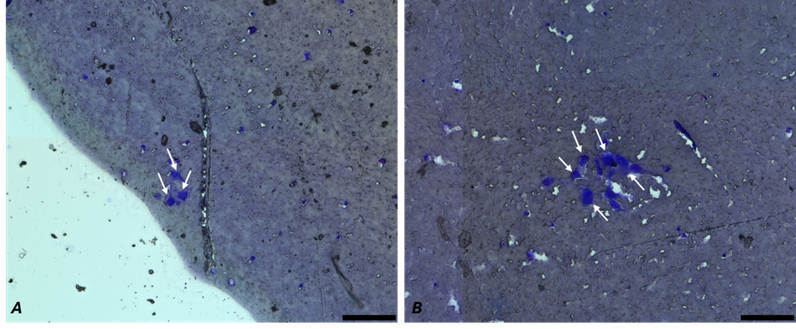
**

**Figure**

**Fig. (A-B). Fine-needle aspirate from a kidney: Diff-Quik–stained cytologic smear. A population of spindle to oval mesenchymal-appearing cells with scant weakly basophilic cytoplasm, marked anisocytosis and anisokaryosis, occasional prominent nucleoli (white arrows), on a haemorrhagic background. Original magnification, 200x. Scale bars, 20 µm.**
